# Academy of Stomatology

**Published:** 1899-11

**Authors:** 

**Affiliations:** Academy of Stomatology


### ACADEMY OF STOMATOLOGY.

THE regular monthly meeting of the Academy of Stomatology was held at the rooms of the Academy, 1731 Chestnut Street, on the evening of April 25, 1899, Dr. M. H. Cryer in the chair.

A paper, entitled "Dental Lesions and their Relation to Nasal and Accessory Cavities," was read by Dr. G. L. Jameson.

(For Dr. Jameson's paper, see page 625.)

### DISCUSSION.

*Dr. Kirk.*—I first wish to express my appreciation of the paper and of the very large number of interesting cases that the essayist has presented. The paper is so full of suggestive points that one hardly knows what to discuss first. There were two or three, however, that caught my attention. He gave importance to the difference in air-pressure in the nasal cavity as affecting the development of the tissues or structures above the nasal chamber. I do not exactly see how a difference in atmospheric pressure, sufficient to bring about a change in the developmental process of these structures, is brought into operation, as I know of no case in which a closed chamber is the result. We have been inclined to attribute this flattening of the sides of the upper arch rather to the pressure of the cheek muscles and loss of pressure by the lip in front as concomitants of mouth-breathing. Dr. Angle has called attention to the fact that the teeth of the lower jaw erupt first, and that these form the mould over which the arch is brought into line. If the jaws are not brought into normal occlusion, the upper alveolar border loses the stimulative effect of the impact of a normal occlusion, and hence there is an arrest of growth, and the teeth do not take their normal places.

The suggestion of the essayist, that pyorrhœa alveolaris may be caused by the irritative effect of secretions which drop constantly over the portion of a tooth impinging upon the nasal floor, is a good one. I should like to have examined that case, to see if there was any other factor which could be drawn into account. I do not at all question the possibility of it, but there are a great many cases of phagedenic pericementitis in individual teeth, in otherwise perfect dentures, caused by a slight irritation from a temporary malocclusion, which becomes greater after a while. Only a few days ago I saw a beautiful set of teeth in the mouth of a young patient, apparently in good health. I made no examination of the nose, but the mouth I did particularly examine, and I diagnosed the case as one of phagedenic pericementitis of an incisor, brought about by malocclusion. The case was operated on to-day for the removal and replantation of the incisor.

*Dr. Jameson.*—I want to state that the first case of irritation of the nose was examined by Drs. Peirce and Kelley. Dr. Peirce said that he could find no probable oral cause for the pericementitis.

*Dr. Cryer.*—I congratulate Dr. Jameson upon his paper, and am glad to know that he has taken so much interest in this line of study. There have been many cases of malposition of teeth in Philadelphia which do not seem to be known to the essayest, among which might be mentioned a case of the late Professor Harrison

Allen, of this city, which was that of a patient whose canine tooth erupted into the nasal chamber, thus separating the septum from the floor of the nose.

One of the most remarkable cases, not heretofore reported, coming to my attention lately, was that of a patient who was referred from the hospital to the dental department of the University of Pennsylvania. The surgeon in charge had cut into the antrum for necrosed bone, and discharged the patient under the supposition that he would get well. The patient returned, and, on close examination, a third upper molar was found with the face of the crown within the antrum, in the upper posterior border of the sinus, and well up under the posterior floor of the orbit. If the roots of this tooth are normal in length and not curved, they must pass through the spheno-maxillary fossa, the apices of the roots thus being in close relation to the body of the sphenoid bone. I have not thought it well to extract this tooth. It appears to be healthy. Those who have studied the anatomy of these parts know how serious it might be to remove this tooth. A larger opening would have to be made into the antrum, and as the roots are in close proximity to the internal maxillary artery, and the great plexus of veins situated in the spheno-maxillary space, there would be considerable danger of hemorrhage.

In regard to anatomical specimens, there are quite a number in the museum of the dental department of the University of Pennsylvania, among them being a supernumerary incisor in the septum of the nose. Dr. Truman has a very fine specimen there, of a canine tooth with the root visible in the nasal chamber. Dr. Kirk also has a beautiful specimen, showing a retarded central incisor lying in the floor of the nasal chamber. When this specimen was found there was a very perceptible enlargement in the floor of the nose, and by removing a thin lamina the tooth was exposed, being accompanied by about thirteen supernumerary teeth, which Dr. Burchard calls "a brood of teeth," representing the incisor as the mother of the brood. I do not know of any case where there were so many supernumerary teeth associated with one normal tooth. It is a most interesting case, and was reported in the *Dental Cosmos* last year. There are many other cases which I might cite.

Dr. Jameson spoke of the inflammation of the mucous membrane of the nasal chamber, and, if I understand him, that it had influence on the teeth.

We not only have that influence on the teeth, but we also have inflammation from the mucous membrane lining the ethmoid cells, and the passage-way extending from the frontal sinus down through the infundibulum and the hiatus semilunaris, and also through the openings from the various cells into this common passage-way. If the hiatus which carries the fluid from the frontal sinus becomes closed by the enlargement of the bulla ethmoidalis, there is only one direction in which the fluid can pass, and that is into the antrum, and as it passes into this cavity it becomes fetid, and causes inflammation that may destroy the nerves and blood-vessels supplying the teeth. I have claimed heretofore, and I believe that I am right, that superior teeth are supplied by a superior dental nerve, similar to that which supplies the inferior teeth. I have many cases and specimens in the University of Pennsylvania from which I can demonstrate that the teeth are supplied by the so-called posterior dental nerve and artery running along the floor of the antrum, covered by the mucous membrane, and giving off branches to the various teeth, then passing forward to the incisor foramina or canals, and supplying the anterior teeth. Now, if the cavity be engorged by fluid, it is bound to interfere directly or indirectly with the nourishment of the teeth situated below, as they receive the nourishment through the floor of the antrum. Hardly a month passes that one does not find teeth loosened, and even lost, by disease, not of the antrum, but of the upper nasal region,—the frontal sinus or ethmoidal cells. This has been proved by the fact that, after the nose has been properly treated, the loosened teeth become firm in the jaw. In one case I had about two years ago, after extracting the first bicuspid and removing necrosed bone, there was a large flow of pus, and by passing a probe into the antrum it went upward out of the antrum into the hiatus, and from the infundibulum into the frontal sinus. This patient subsequently passed out of my hands and was admitted into the medical department, where she was treated for meningitis. When the explanation was made to those in charge that it was possible that the diseased track spoken of was the cause of the meningitis, she was sent again to the dental department; the parts were thoroughly opened and curetted, and the patient restored to health. A great many of our rhinologists and dentists believe that disease of the antrum is caused principally by the teeth. Even the late Professor Harrison Allen claimed that three-fifths of antral trouble was caused by teeth. I do not think so. I believe that more teeth are lost by disease of the nasal chamber and its associated cavities.

The doctor spoke of a "spur" of the septum passing over to one side of the nasal chamber and thus almost closing it. Dr. Kyle, I believe, is producing an illustration, in his new work, from one of my specimens, where there is no opening left through the inferior meatus, as a great "spur" passes over and presses against the inferior turbinated bone. The inspissated matter deposited in this position may create an irritation, and through this irritation disease or necrosis of the bone may take place.

The doctor also spoke of a case of a third upper molar being passed out of the nose, the route of the passage being from the place of development through the antrum, then into the nasal chamber, and out of the nostril. I think it is more than likely that, in this particular case, the antrum was small and its floor high up, the nasal chamber passing from the septum to the outer wall of the maxillary bone. I have several specimens of this character. Dr. Kyle shows an illustration where the roots of the molar are visible in the nasal chamber. The floor of the antrum being on a higher plane and above the floor of the nasal chamber, the latter extends to the outer wall of the maxillary bone. In a skull of this kind the teeth could very easily be erupted into the nasal chamber, and having no occluding tooth to oppose it, it could "elongate" until it became free in the nasal chamber, then pass into the pharynx or out through the nostril.

The doctor spoke also of using peroxide of hydrogen. I seldom use this medicament, especially in abscesses of the jaw. On superficial surfaces or in cavities which have large openings and will allow the easy exit of decomposing pus and gases it is valuable, but in small, closed cavities, without sufficient external openings, it is liable to do much damage by producing pain and dissecting up the tissue covering the fine air-cells. In the lower jaw it may enter the cancellated tissue or the inferior dental canal, dissecting the tissue from the bone; and, I believe, it can force germs in advance of it and drive them into positions that they would not otherwise reach.

In a recent case, at the University of Pennsylvania, the patient, a woman about twenty-five years of age, had an upper right second bicuspid tooth which became devitalized, and dento-alveolar abscess formed. Her dentist had injected peroxide of hydrogen through the tooth into the surrounding tissue and fistulous opening. The gum tissue was stripped from the bone, from about the centre of the first bicuspid to the centre of the first molar. The bone died, forming a sequestrum extending from the canine tooth to the second molar, and from the border of the alveolar process to a little above the level of the floor of the antrum. This was finally removed.

Peroxide of hydrogen, when kept, may become stronger. In this condition, I believe, it will cause necrosis of the bone more readily by destroying its nutrient vessels than through its escharotic power. It is possible first to destroy the trophic nerves, and in this way cause necrosis of the tissue.

*Dr. Peirce.*—I have been very much interested in the paper and in the variety of cases that have been enumerated; all have been of great interest. A case was brought to me from Reading by Dr. Tait some two weeks ago. The patient had an abscess in the palate, also one upon the buccal aspect of the alveolar process, occupying a considerable space over the region of the first bicuspid. On pressing the abscess in the palate decided fulness over the region of the bicuspid was noted, and *vice versa.* The first bicuspid contained a devitalized pulp. I suggested the removal of the tooth; the patient consented; the doctor also thought it wise, and on removal I found that the tooth had two decided roots. But, to my great surprise, I found that each root had penetrated the antrum. The abscesses on the palate and buccal surface were not relieved in the least. Fluctuation was felt distinctly, but there was no discharge from the alveoli. On placing my probe up into the socket, I found a septum of bone that had filled the space between the two roots. I drilled out the bone, and the moment I went through it the pus hissed out forcibly into the patient's mouth. It was surprising to me that those two abscesses should unite through this little septum of bone that passed between the roots, and the doctor assured me that he had treated these roots and that the tooth had not been sore. I questioned the patient as to whether he had any antral trouble. He said not, but that on one or two occasions he had found a little discharge on his pillow, which he thought came from the nose, but the nose was perfectly clear. There must have been a small opening from the palatine abscess into the antrum that produced a slight discharge, so slight that it could hardly be noticed. It was an interesting case to me.

The case I had the pleasure of seeing with Dr. Jameson was of great interest from the obscurity of its cause. There had doubtless been some previous irritation with which, by continuity, it had been associated. Another somewhat similar case I have had, with the dentist in charge, under my care. The last time the patient came in I found near the apex of the root a little roughness under the mucous surface. I made an incision into the gum with my lance and took out quite a large piece of necrosed bone; but I could not discover in Dr. Jameson's case any necrosis. Its obscurity made it interesting, and I was glad to hear the doctor's explanation of its cause, which he thinks he has discovered.

*President.*—Dr. D. Braden Klye, of the Jefferson Medical College, is with us to-night, and I think we would all be pleased to hear from him. He does a great deal of work in this special line, and, with your permission, I will call upon him.

*Dr. D. Braden Kyle.*—Mr. President and Members of the Academy: I have been especially interested in the paper and in the discussion. As there were a number of points raised and a number of questions asked, I want to say a few words in regard to nasal obstruction, as described by Dr. Jameson. I would like to ask Dr. Kirk in regard to the cases, whether or not the isolated cases of pyorrhœa are not in a direct line with the nasal cavity?

*Dr. Kirk.*—Some were in the lower jaw.

*Dr. Kyle.*—Are the majority?

*Dr. Kirk.*—I could not say; I think not.

*Dr. Kyle.*—The reason I ask the question is, my attention was called to the condition of the septum, which was not a spur, but a redundancy of tissue, a condition which, if the septum be straightened, there is too much tissue, and it would elevate the nose, and for that reason is crowded down on one side of the septum, almost in contact with the floor of the nose. In every case in which that cause existed there was something wrong with the teeth. At present I have three cases in the clinic in which there is sinus-formation. One case was operated on at another hospital for antral lesion. The antrum was opened, but nothing was found to be wrong. Nevertheless, on examination of the nose this condition was present, and on passing the probe we could not enter the cavity, but on using methyl blue, the blue appeared in the nostril, although we were not able to pass a probe of soft wire clear to the nostril. There was a sinus from the floor of the nose directly underneath, such an obstruction as described by Dr. Jameson. In one case it was in the right nostril; in the other, in the left. It looks as though that irritation of the septum bore some etiological relation to the diseased condition of the tooth, and I think in some cases it explains the cause. In the early stage, the inflammation in the nose would set up an inflammation in the nasal mucous membrane of the floor of the nose and accumulations underneath the redundancy of the septum. That irritation, if the upper jaw were thin, would affect the blood- and nerve-supply of the teeth, and inflammatory swelling would force the tooth down. I have a patient now in which the front tooth is directly under a projection from the septum which extends close to the floor of the nose on that side; the tooth is so loose you could pull it out; it moves back and forward. It is in such a condition that it may drop out at any time, and the dentist has a plate made ready to fit in a tooth. In that nostril he has had for years almost complete occlusion.

The point raised by Dr. Kirk in regard to air-pressure is an interesting one. How much effect actual air-pressure has in a nostril is a question. But the fact remains, if you take the trouble to examine your patients, you will find in a majority of cases that in the individual with a wide-open nostril—a nostril which will extend, so that the patient breathes freely—there is a perfectly formed septum and a regularly formed upper jaw, and that the individual who has a worm-hole nostril has irregular teeth; these two conditions go together. How much atmospheric pressure has to do with it I will not say; but this is true, that the pressure largely is counteracted somewhat by nasal breathing,—free nasal breathing. Within the nose there is no moving current of air to resist pressure. On the other hand, the current of air passes through the nose and stimulates to action the membrane that has a blood-supply, and that action brings nutrition to that membrane, and nutrition to that membrane means development; but that does not cause narrow nostril, because in many cases in which you have obstruction there is hypertrophy of the turbinates, enormously thickened membrane. Yet the nutrition is unquestionably lessened in these cases, and that would cause irregular formation or maldevelopment. I think the solution lies in a number of causes. Whatever the solution may be, I think you will all agree that wherever you find nasal obstruction you will find irregular upper teeth.

I agree with Dr. Cryer in regard to the use of hydrogen peroxide. It surely changes in strength, and at times it is an escharotic; it has the same action as strong nitrate of silver or chloride of zinc.

The condition described—as shown in a cut made by Dr. Cryer—in which some of the diseased teeth punctured the antrum, in which there was no infection, and in which there was associated ozæna, is really an interesting condition.

I think the rhinologists are really more closely related to the stomatologists in their profession than are any other specialists, and that the rhinologist either should be a good dentist or that those cases should fall into the hands of dentists, because there are so many conditions of the floor of the nose and antrum, and all the accessory cavities related to the teeth, that require special knowledge. If there is anything that drives a patient to a doctor it is an offensive catarrh. Treatment of the nasal cavity, then, is only keeping up a nasal irritation, while the trouble is, in reality, in the antrum. If the irritation in the opening of the nose is such that gas will accumulate in the antrum, you will have all the symptoms of confined suppuration,—it is really not ozæna at all, in the true sense of the term; it is really an odor. Such a case belongs to the dentist, not to the rhinologist. The trouble is caused by the tooth.

I would like to emphasize this point, that many of the intranasal and accessory cavity lesions could be entirely avoided by careful attention to nasal breathing in early life.

*Dr. Kirk.*—Before Dr. Kyle leaves, I would like to say a word. When I referred to the question, I was not able to understand the difference in atmospheric pressure. I did not mean the question of impact of the atmosphere against that membrane, but I spoke of the difference in atmospheric pressure as we speak of the difference in hydrostatic pressure or in electricity of the potential. The case of the occlusion of the tooth is the condition I have supposed these rhinologists were referring to when they speak about pressure in the nasal cavity. Under what conditions can we get a state of affairs in the nasal cavity analogous to that which we have in occlusion of the Eustachian tube? I cannot understand the difference in atmospheric pressure, as I use the term, to occur in the nasal passages by any pathological condition that I have heard of. It may be due to some cause for which the word "pressure" is a misused term. But I have heard it used by rhinologists, or at least those who consider themselves rhinologists, in the sense of hydrostatic pressure. It is an unconceivable thing, as I understand, although imperfectly, the anatomy of these parts. I want to repudiate the idea that I was taking exception to the explanation of the etiology of this difficulty. I was glad to learn of it. It was quite in line with other conditions of that sort, as reported by Dr. Cryer, but I only raised a query, so that it should not be accepted as an explanation of all of these cases of individual phagedenic pericementitis, as they occur in the lower as frequently as they do in the upper.

*Dr. Curry.*—Apropos of the atmospheric pressure question, I

had a case recently of a palate cleft to such an extent that the nose and oral cavities were practically one. It was a case of mouth-breathing, but in that case I noticed, as Dr. Kyle stated, that all the turbinated bones were hypertrophied to such an extent that they almost filled the nasal cavity, while the nostrils were so hard and so small that I could barely get a lead-pencil through them. This case seems to show that atmospheric pressure does not produce that kind of a nostril, or condition of the membrane, because the mouth and the nose in this particular case were a continuous cavity.

*Dr. Register.*—In support of this exceedingly interesting paper I desire to state that a patient of mine was seized with facial neuralgia of a very severe character. Not getting any relief at the hands of his physician, he came to me. Examination brought to light the fact that he had never erupted the left cuspid tooth. There was a very small fistulous opening in the cuspid region, and the tooth was evidently lying horizontally across the jaw, with the apex pointing towards the ala of the nose. He had no nose or other complication. Under nitrous oxide I burred away the bone and lifted the tooth from its position. When it came away there seemed to have been no bone tissue between the ala nasi and the root apex. I carefully examined the tooth at the time and found quite an incrustation, now explained as a deposit of serumal calculus. The removal of the tooth put an end to the neuralgia.

Another patient has been suffering for some little time with a cutaneous trouble over the malar bone; the left side of the face being slightly enlarged. I noticed that he had some little necrosis, apparently of the jaw, from a devitalized tooth, upon which he had worn a crown. It gradually became so affected that I took it out, and the pain on the face was so severe that he could hardly touch it with his finger or a handkerchief. Some three or four days ago I went into the antrum, in a very limited way, and got no sign of any accumulation there. I then bored along the bone, which was more or less soft and I think necrosed. I treated it with sulphuric acid, fifty per cent., and afterwards packed it with a sterilizing preparation, and let it remain until it came away. I was very much pleased to get a postal from him yesterday, telling me that he was absolutely relieved from pain.

*Dr. Brubaker.*—I have listened carefully to the remarks regarding stenosis of the nasal chamber and irregularities of the dental arch, as well as remarks relating to atmospheric pressure. While it is possible that stenosis and difficult breathing may stand as a cause of dental irregularity, I think it is quite possible, as Dr. Peirce has suggested, that the two are simply the effects of some underlying cause; that the defect in the development of the nasal chamber is only a part of a defect, seen also in the superior maxilla. I confess that I cannot understand how any variation in pressure or difference in pressure could cause any defects of the septum. Atmospheric pressure is practically a standard thing, as long as there is any communication with the external atmosphere. If there is a stenosis of the left nasal chamber, the air passes very freely into the right; and if there is but a very slight opening in the left nasal chamber, the air will also pass through that opening. The movement of the air through the nasal chamber is not due to any exertion of the mouth-apparatus, but is caused by contraction of the chest walls and diaphragm. They are the agents that lower the pressure in the interior respiratory passages, and it is this that makes the external pressure, driving the air into the nasal chamber and into the respiratory passages. It does not make any difference whether the nasal chamber is small or large. The air goes through both chambers, but it is not unequal; there can be no variation of pressure in those chambers, if there is any communication. I do not see how it is possible for atmospheric pressure in a healthy chamber to act as a pressure against a septum of the nose.

*Dr. Jameson.*—I will only say what I should have added to my paper, that all dentists should examine the mouths of their patients and make a point to investigate whether their mouth-breathing apparatus is normal or not. A patient first comes to the practitioner and then to the dentist. It is the former's duty to save the child a great deal of future trouble by proper directions.

Otto E. Inglis,
*Editor Academy of Stomatology.*

